# Context matters: A review to formulate a conceptual framework for coaching as a disability accommodation

**DOI:** 10.1371/journal.pone.0199408

**Published:** 2019-08-22

**Authors:** Nancy Elizabeth Doyle, Almuth McDowall

**Affiliations:** 1 Department of Psychology, City, University of London, London, United Kingdom; 2 Department of Organizational Psychology, School of Management, Birkbeck, University of London, United Kingdom; The University of Memphis, UNITED STATES

## Abstract

Although dyslexia affects 5–8% of the workforce this developmental disorder has not been sufficiently researched in adult populations. Yet a diagnosis confers legal protections as employers must provide disability ‘accommodations’ to assist work functioning and performance. The implementation of such accommodations, including coaching, lacks theoretical framing and evaluations of impact in practice. Recognizing a need for conceptual work, we undertook a narrative, systematic scoping review from a realist pragmatic epistemology, taking an iterative approach to define and address the review question: ‘to what extent, and under what conditions, can face-to-face learning interventions improve Working Memory (WM) and Self-Efficacy (SE) and can these lead to functional improvements related to work performance?’ Informed by expert and stakeholder consultation and user data, our review extracted and synthesized 25 studies from eleven countries to identify potentially applicable learning intervention theories, their effects upon WM and SE but also functional outcomes such as comprehension. We suggest that intervention protocols informed by Social Cognitive Learning Theory can improve SE, as would be expected, and more surprisingly also WM. The development of metacognition, stress management and fidelity to Goal Setting Theory were identified as valuable intervention features. We propose that coaching activities may provide a more contextualized environment for transfer of learning from WM to functional skills such as comprehension, when compared to computerized training interventions. We call for theoretically underpinned, primary studies to evaluate interventions with adult dyslexic populations to further our understanding of disability accommodations.

## Introduction

Developmental disorders, including dyslexia, are common in adult working populations affecting about 5–8% of the workforce [1] and are subject to relevant disability employment legislation in many advanced economies including the UK, the Netherlands, Canada and the USA [2–4]. Yet understanding of targeted support to facilitate career success and well-being for affected individuals remains under-developed. The Americans with Disabilities Act [5] and UK Equality Act 2010 [6] require employers to make ‘accommodations’ (US terminology, which we adopt for the purposes of this paper) or ‘adjustments’ (UK terminology) to ensure that workers are not disadvantaged. The UK act refers to a range of protected characteristics to define disability: dyslexia is often referred to as a ‘hidden’ or ‘invisible’ disability as characteristics are not necessarily apparent to the untrained eye [6], [7]. In the UK over 3,000 individuals per year each receive approximately $1,000 of public funding to access support, including assistive technology and coaching [8–10] to facilitate relevant accommodations. Although coaching, defined broadly as working with dyslexic individuals on a one-to-one basis to help them with work issues, is considered a cost-effective investment of public funds [10], there is much variability in delivery style and quality [11].

However, work-relevant extant dyslexia literature is limited to disclosure [12], [13], bias in the interpretation of legal obligations [3], [14], [15] and the barriers presented in understanding relevant activities for employees with dyslexia [16], [17]. Therefore, theoretical framing for the purported effectiveness of coaching or other accommodation activities is lacking. Rice and Brooks ([1], p. 12] stated in 2004: “*good practice in this field rests almost entirely on professional judgment and common sense*, *rather than on evidence from evaluation studies”;* we contend that it is now urgent to guide practitioners and policy makers towards evidence-based interventions [6], [12] for disability accommodations. The principle purpose of this review is to scope a ‘blind spot’ [12], [18] in psychological research through a Narrative Systematic Review and, using inductive reasoning [19], develop a theoretical explanation of “how and why programs work” ([20]p. 74), to identify mechanisms from relevant interventions that could conceivably be applied to an adult dyslexia coaching context [21–23]. Key to our approach was the consideration of cognitive and psychosocial factors to inform future dyslexia research, given that previous reviews have highlighted insufficient attention of these for adult populations [2], [24]. We now turn to some theoretical considerations relevant to our review protocol and also highlight inconsistencies in international practice, with reference to socio-legal context which is often neglected in psychological research.

### Dyslexic adults: Theoretical considerations and workplace outcomes

Historically, dyslexia was thought to be a visual processing disorder often termed “word blindness” ([25]. Current dominant theory in developmental and educational research refers to a deficit in phonological processing [26–28]; however, rapid-naming (visual recognition of words at speed; Denckla & Rudel [29]) still informs UK diagnosis [30], [31]. The double-deficit hypothesis [32]), which refers to difficulties in rapid-naming and the accurate decoding of sounds, is therefore still used to define and determine individual cases [31]. In the USA, eligibility for support is benchmarked by criteria set by the Individuals with Disabilities in Education Act, such as reading age [33]. This is also the case in Australia where practitioners rely on a generalized low achievement model for identifying individuals in need of support [34]. We thus identify a lack of consistency to diagnostic criteria depending on geographic location [35–38] which impedes broader understanding of how individual cases may (a) be affected at work and (b) qualify for disability protection in employment.

Neuropsychological researchers have highlighted ‘working memory’ (i.e. our capacity to hold information in our attention and manipulate it [39]) as a potential primary neural deficit underlying phonological processing difficulties, contributing to delayed literacy acquisition [40–43]. Other potential causal mechanisms include different long-term memory-based hypotheses, such as the cerebellar deficit theory, which focuses on a lack of automatization and issues with balance/motor control [44]. Interestingly, international neuro-imaging comparisons suggest that the neuropsychological structure of dyslexia may vary according to the language that one speaks [45], [46]. This points to the potential interaction of neural and situational as well as cultural influences, the influence of context. For example, for visually constructed languages such as Chinese, visual processing strengths are key for literacy whereas for sound-based languages, phonological processing is key. Yet some researchers remain unconvinced that any neuropsychological features of dyslexia are sufficiently distinct to differentiate from general poor reading skills [47], [48]. Given such disparate views on etiology and symptomatology, any research in the field must be considered nascent rather than grounded in evidence.

This notwithstanding, the necessity for disability accommodations for dyslexic adults in organizational practice is evident when considering typical levels of occupational exclusion and the potential impact on individuals with wider social costs. Dyslexia is associated with higher rates of criminal activity [49], higher unemployment [50], failure to achieve potential post-education [51] and impaired workplace participation defined by de Beer et *al*., ([2]; p. 4 [52]) as “work content, work circumstances, terms of employment and relationships at work”. According to one study, only 1% of corporate managers are dyslexic [53] compared with a population norm of 10% [54], demonstrating an unequal pattern in career progression. The overarching issue of (lack of) social inclusion and equality [55–57] creates a moral imperative to ensure any accommodations are substantiated by evidence and guiding theoretical frameworks. Yet understanding of adult symptomatology remains underdeveloped, not least because research relies heavily on correlational data and designs [24]. Doyle and McDowall [58] report only 41 occupationally-focused studies on dyslexia out of an initial search return of over 11,000 and only 800 of these concerned adults at all.

### The review aims

Given the worldwide call for clear guidance on how to operationalize disability legislation for invisible disabilities [12], [16], this review aims to: (a) elicit psychological mechanisms and accommodation activities of relevance to the workplace for the dyslexic adult through an initial scoping process based on practitioner advice, stakeholder considerations and contemporary research in order to; (b) propose a hypothetical intervention pathway, upon which to build a research agenda for future intervention studies.

## Method

Our extended method section details our process for scoping the parameters required for a narrative systematic extraction, as well as an iterative further review of the literature as commensurate with an inductive approach.

### Scoping the research question: Expert panel consultation and practitioner data review

Given our context-sensitive realist epistemology, we drew on the context, intervention, mechanisms and outcomes (CIMO) framework recommended by Denyer and Tranfield [59] starting with a scoping review to inform our protocol. We consulted a virtual panel of 8 internationally-recognized dyslexia experts, including academically-published educational, occupational and clinical psychologists, educational neuropsychological researchers and one dyslexia specialist from the charitable sector. Three dyslexic employees and three employers of dyslexic people in the interview sample to embed the perspective of the end user in the scoping review [60–62]. Semi-structured interviews included questions about (a) frameworks or evidence for supporting dyslexic adults through accommodations; (b) current thinking on symptomatology of dyslexia and how relevant these are to adult populations; (c) which research questions should guide this review; (d) the direct identification of primary literature relevant to this review process; and (e) priorities for future research. The interviewees’ responses were recorded in note form, retaining verbatim answers where possible. Following guidelines for applying methodological rigour to qualitative, inductive analysis [63], the interview notes were reviewed to identify preliminary patterns and commonalities, revealing that answers coalesced in three main areas: (a) nature of dyslexia in adults; (b) specific issues pertaining to adult populations and (c) the type of research needed. The interview notes were then reviewed further to catalogue excerpts and phrases by similarity and counted to weight responses. The primary researcher conducted the initial analysis which was cross-checked with the second researcher to ensure this captured all salient issues from the interview data. Any areas of ambiguity were resolved by returning to the original notes and subsequent discussion, consulting the panel on the interpretations and findings during the iterative extraction to provide a third-party check on process.

### Panel data

The data indicated a lack of studies directly concerning themselves with coaching interventions for dyslexic adults, which led us to include studies in the full review from different samples (child dyslexia or adult populations with other conditions) so long as content was relevant to address the review questions. The data confirmed that coaching activities are routinely prescribed as a disability accommodation for employees in the UK and that two core psychological constructs appear crucial to coaching success. Accordingly, we created a hypothetical framework for evaluation of (1) face-to-face intervention evaluations (as opposed to computerized training or e-learning, congruent to UK accommodation practice) involving (2a) working memory (WM) and (2b) self-efficacy (SE) as target-intervening variables. Table 1 summarizes our interpretation of the CIMO framework, explaining how we defined and operationalized the relevant stages throughout the review process, from initial definitions and consultation processes through to the data coding and extraction.

**Table 1:** Interpretation of the CIMO framework for the current realist synthesis.

Realist synthesis component	Explanation (Denyer & Tranfield, 2009)	Relevance to the present review	Addressed in the review protocol through?
Context	• Individuals of interest. • Interpersonal relationships of interest. • Institutional setting of interest. • Aspects of wider infrastructure of interest.	• Realistic approach to lack of context-specific studies for WM. • Include adults and children. • Include both men and women. • Include all nationalities. • Consider educational, health and occupational contexts.	• Consultation of experts. • Consultation of ‘end users’. • Review of practitioner data.
Interventions	• The intervention of interest.	• *Exclude* medical- or technology-based memory training interventions. • Include interventions described as ‘learning’ or ‘coaching’. • Include interventions delivered via face-to-face dialectic pedagogy. • Exclude didactic-type interventions.	• Consultation of experts. • Search protocol and search terms. • Inclusion and exclusion criteria. • Data extraction.
Mechanism	• Mechanisms of interest. • Explanation of how the interventions act within the context to lead to the outcome. • Understanding of how mechanisms are activated or not activated in different contexts.	Given that the distinction between mechanisms and outcomes is in reality often blurred, WM and SE were both considered mechanisms and outcomes in the search for studies, provided that the study included a standardized measure of either mechanism as a dependent variable.	• Consultation of experts. • Further iterative literature review. • Coding of variables during data extraction.
Outcome	• Relevant outcomes. • Measurement of outcome. • Primary and secondary outcomes.	Additional mechanisms (contributing to WM/SE improvement) and work-related outcomes, such as higher order reasoning abilities, were also considered in the extraction and synthesis.	

Regarding symptomatology, only 18% of responses referenced literacy difficulties in adults yet 51% of responses made direct references to Working Memory (WM) or indicators of WM-related behaviors [64]. WM has been defined as “assumed to be a temporary storage system under attentional control that underpins our capacity for complex thought” ([65] p. 1). The employees and employers did not use the term, but reported functional difficulties associated with WM [64], such as “following instructions”, “keeping up with conversations” and “in meetings remembering what people have said”. Psychosocial difficulty was the second most frequent response (27%), highlighting the need to support Self-Efficacy (SE) beliefs. SE refers to the development of our perceived ability to perform a given task and is a central tenet of Social Cognitive Learning Theory (SCLT, [66]). The entire panel stressed the need for evaluations of current practice, with 45% drawing out the need for clarity on coaching interventions.

As an additional reference point for grounding our review in pragmatic as well as conceptual and empirical considerations, we also reviewed survey data collected by the British Psychological Society from working dyslexic adults (*N* = 81[67]). Results documented that the most frequent focal topic of occupational disability accommodation requests was memory support, as highlighted by 71% of respondents. Although literacy issues persist into adulthood for people with dyslexia [68], these were not seen as the primary workplace concern by experts or stakeholders and may well have been adequately addressed through earlier educational interventions or assistive technology [69].

In conclusion, congruence between expert panellists, stakeholders, practitioners and preliminary research suggested that WM and SE are the most important psychological constructs to target when providing occupational support for adults with dyslexia; an interesting finding in and of itself, given the stereotypical literacy-based definitions [70]. Support for this position is present in practitioner literature. Coaching is recommended widely by dyslexia practitioners in the UK for a variety of purposes and contexts, including education and work [7], [71–73]. A pilot study of dyslexic employees and supervisors [74] focused on coaching effectiveness. The study highlighted memory issues as the most prevalent focus for coaching and SE as a potential construct for further investigation given the importance and salience of psychosocial factors in the workplace (literacy issues were in fourth place of priority in this study). Both variables were reported (self and supervisor dyads) to improve following coaching at a three-month interval. Other qualitative and cross-sectional research has also pointed to low levels of SE as particularly problematic for dyslexic adults [4], [75] associated with the likelihood of a range of difficulties, including cognitive working memory [76].

We undertook a further, iterative literature review based on the outcomes of our initial scoping consultation, and now briefly outline both constructs and their relationship to work performance.

### Working memory and work performance

WM is linked to effective cognitive processes [42], [77–80] and relevant to a range of effective work-related behaviors, such as self-regulation [81], time management [82] and management of complex environments [83]. Although the association between WM and work performance provides a strong rationale for targeting relevant supportive interventions, the question of *how* WM could be improved through coaching remains unanswered. WM intervention research has a growing body of literature on adaptive computerized training, yet there is lack of clarity concerning causal pathways for lasting WM improvements. Systematic reviews of computerized interventions demonstrate that when WM is targeted it improves (termed ‘near transfer’); but these improvements often fail to translate to wider successes in complex reasoning (termed ‘far transfer’) across a wide range of client groups including: children with reading disabilities [84]; clinical neuropsychological rehabilitation [85]; and healthy populations, [86]. Given that successful transfer to improved workplace performance is a necessary prerequisite for disability accommodation, we therefore cross-referenced cognitive WM improvements with functional measures, such as reading comprehension, in our stage two extraction. More specifically, we aimed to explore the extent to which coaching interventions that improve WM may also result in improved work performance-related, functional outcomes.

### Self-efficacy and work performance

De Beer et *al*.’s [2] systematic review of factors influencing work participation for dyslexia acknowledged many potential psychosocial mechanisms, including cognitive (WM), but also emotional, behavioral, social and environmental factors. Functioning and performance in employment contexts are reported in general populations to be contingent on supportive interactions with others and on positive self-belief [52]. A seminal meta-analysis by Stajkovic and Luthans [87] showed that (high) SE, similarly to WM, has a strong relationship with work performance in the general population. Indeed, SE has been researched far more frequently than WM in industrial, occupational and management psychology [88–91] as high levels are related to improvements in occupational outcomes for people with mental health needs [92] and chronic conditions [93]. In particular, high SE is reported to maximize career potential for dyslexic adults compared to those with low SE [3], [94].

#### The working memory and self-efficacy relationship

Empirical evidence from aging populations [95] demonstrates that SE potentially moderates WM-related performance deficits [76]. The ‘Gerber-Leather model’ [2] posits that for dyslexic adults: (a) the self-regulation of memory (WM) and (b) positive reframing of the individual’s personal experience act as mediators of an improved sense of control, in turn influencing success in the workplace. Consistent with the data from the expert panel, we note persistent references to the importance of SE and WM in the limited literature on dyslexic adults [12], [75], indicating links between these two psychological mechanisms and work performance outcomes. We now turn to coaching as an activity to improve functioning in a disability context.

### Coaching for dyslexia

Coaching psychology literature defines workplace coaching as a developmental activity for individuals and organizations [96] aiming to unlock an individual’s potential [97] and ultimately improve workplace productivity and job performance. Coaching is reported to help individuals work on cognitive, behavioral and emotional changes rather than simple knowledge transfer, supporting the need for a more dialectic than didactic pedagogy [98], [99]. McLoughlin and Leather ([71] p. 43) describe workplace dyslexia coaching as an “androgogical approach” that relies on the metacognitive experience of dyslexic adults [76]; they do, however, highlight that deviations from this style are common in practice.

In contrast to the various tuition interventions provided in education [100], [101], which focus on study and classroom skills to facilitate literacy and spelling, coaching as commonly used in UK disability accommodation [7], [102] focuses on outcomes more commonly associated with underlying WM issues, such as time management and organizational skills, [58], [71] to help optimize work performance and functioning. Workplace coaching objectives are therefore potentially better suited to adult dyslexia disability accommodation than literacy improvement targets.

Coaching intervention studies report success in improving occupational difficulties associated with neurological trauma [103]; autism [104], [105] and Attention Deficit Hyperactivity Disorder (ADHD; [106–108], sometimes directly referencing improved cognitive capacity including working memory as the mechanism. Given that relevant studies are typically concerned with work preparation in education, they lack a conceptual framework to explain *how* coaching around cognitive skills may lead to either occupational inclusion or work-related symptom improvement. Conversely, coaching psychology literature reports wide success in improving SE in workplace contexts as a frequently deployed outcome measure with conceptual framing leading to functional performance outcomes [109–111]. Thus we prioritized synthesis of relevant intervention features from relevant primary studies which could be mapped into a dyslexia accommodation protocol.

We further noted the differing learning experiences between a coaching psychology intervention based on self-directed, yet conversational, social learning protocols vis-à-vis computerized WM training, which is a solitary, technology-based exercise, practicing similar tasks repetitively. We compared our extraction results to computerized training in the discussion to consider the respective merit of both interventions in disability accommodation settings.

### Refined research question

Based on the iterative and extended scoping stage of our research, the primary question guiding the second stage of our review was: to what extent, and under what conditions, can face-to-face (C) learning interventions (I) improve WM (M_1_) and SE (M_2_) and can these lead to functional improvements related to work performance (O)?

### Context and intervention extraction

We limited inclusion to English Language. During initial searching and screening we included all face-to-face interventions, as defined by the absence of technology rather than the inclusion of coaching in particular, to facilitate later comparisons of the WM extraction to systematic reviews of WM computerized training interventions. For WM, we included all contexts and populations (healthy or not, education- or work-based) with the exception of: (a) samples with serious age-related cognitive impairments (e.g., dementia) due to the multiple cognitive, social, health and clinical concerns and; (b) child-based studies where interventions focused solely on the actions of the parents or teachers. A more mature research field allowed us to focus on adults in employment for the SE extraction.

We analyzed all extracted studies regarding the extent of their fidelity to a dialectic, recognized coaching definition (see McLoughlin and Leather [71]) consistent with prior studies for other hidden disabilities, including ADHD where affected individuals also exhibit poor memory skills and low self-concepts [108], [112].

### Mechanism and outcome extraction

We excluded studies that did not include WM or SE as a clear mechanism or outcome, for example when measured as an independent rather than dependent variable.

#### Working memory

We identified WM-focused studies through a protocol of a published, standardized WM test (including computerized versions such as *n*-back) as a dependent variable. While this limited the number of eligible returns, this inclusion was deliberate to avoid over-reliance on self-report measures / qualitative research and to draw out studies using reliable, objective measures that were less likely to be influenced by participants simply enjoying their experience [113]. We extracted functional, work-related outcomes (for example comprehension) as secondary dependent variables to address the need for occupationally-relevant performance improvement as required for disability accommodation (currently reported as less successful ‘far transfer’ in computerized WM research [85]).

#### Self-efficacy

We identified SE-focused primary studies through (a) the explicit use of the term, (b) reference to SCLT, and (c) use of a validated SE scale as a dependent variable. Other measures of work-related functioning were reviewed as secondary outcomes for consistency with the WM analysis; these were not considered further in the synthesis given that they were entirely consistent with previous SE coaching research [110], [114], [115], thus offering no further theoretical insights.

### Search criteria

Mapping the search terms as confirmed by the expert panel against the CIMO framework, as depicted in Table 2, our search used EBSCO-hosted databases in June 2016, identifying 609 studies for WM and 414 for SE from Academic Search Complete; Applied Science and Technology Source; British Education Index; Business Source Complete; Child Development & Adolescent Studies; CINAHL Plus with Full Text; Communication Source; Criminal justice Abstracts with Full Text; Education Abstracts (H.W. Wilson); Educational Administration Abstracts; Education Resource Information Center (ERIC); Health and Psychosocial Instruments; Health Policy Reference Center; Medline Complete; PsycArticles; PsycINFO; SocINDEX with full text; Teacher Reference Center. The first author has maintained alerts on academic platforms (RefWorks and Research Gate) to capture any new studies meeting the criteria and, as of October 2018, no new studies have been added.

**Table 2:** Search terms.

CIMO stage	Search terms (using * to denote multiple possible endings to the word)	Search location or stage
**Context**	Coaching OR training OR classroom OR professional development OR intervention OR activity OR learning OR face-to-face OR tuition OR educat*	All Text
**Primary context of interest**	Dyslexi* OR adults OR 19+	Filtering term applied after the initial search to identify high relevance studies
**Interventions**	Learning OR metacognitiv* OR self-awareness OR self-development OR synesthe* OR synaesthe* OR instruct* OR knowledge OR personal development	All Text
**Mechanism / outcome 1**	Working memory OR executive function* OR attention OR short-term memory OR cognition OR metacogniti* OR time management OR self-regulation OR synesthe* OR synaesthe* OR mental function*	Title / subject / abstract / keywords
**Mechanism / outcome 2**	OR self-efficacy OR perceived self-efficacy OR work efficacy OR self-efficacy belief OR social cognitive learning theory OR social learning theory OR self-esteem OR self-confidence OR participation OR social interaction OR agency OR career agency	Title / subject / abstract / keywords

### Abstract filtering

We exported the search results into a bespoke data extraction form to screen all abstracts for relevance to the review questions using ‘2’ for highly relevant; ‘1’ for possible relevance; and ‘0’ indicating not relevant. The first author undertook the first review; all articles in the ‘possible’ category were also reviewed by the second author. In line with our pragmatic epistemology, this was undertaken through a process of clinical judgment and consensus, rather than blind rating (commonly associated with positivist stances). Where relevance could not be assessed in the abstract, full papers were read with a view towards inclusion rather than exclusion if uncertain. During this initial screening, we excluded studies relying on (a) Transcranial Magnetic Stimulation (rTMS) interventions; (b) computerized training only; (c) non-intervention studies (such as correlational designs); (d) those with different types of memory (not WM); (e) Self-esteem or confidence instead of SE; (f) not a face-to-face intervention; (g) no description of the intervention content and (h) target variables researched as independent, rather than dependent variables.

**Fig 1:**
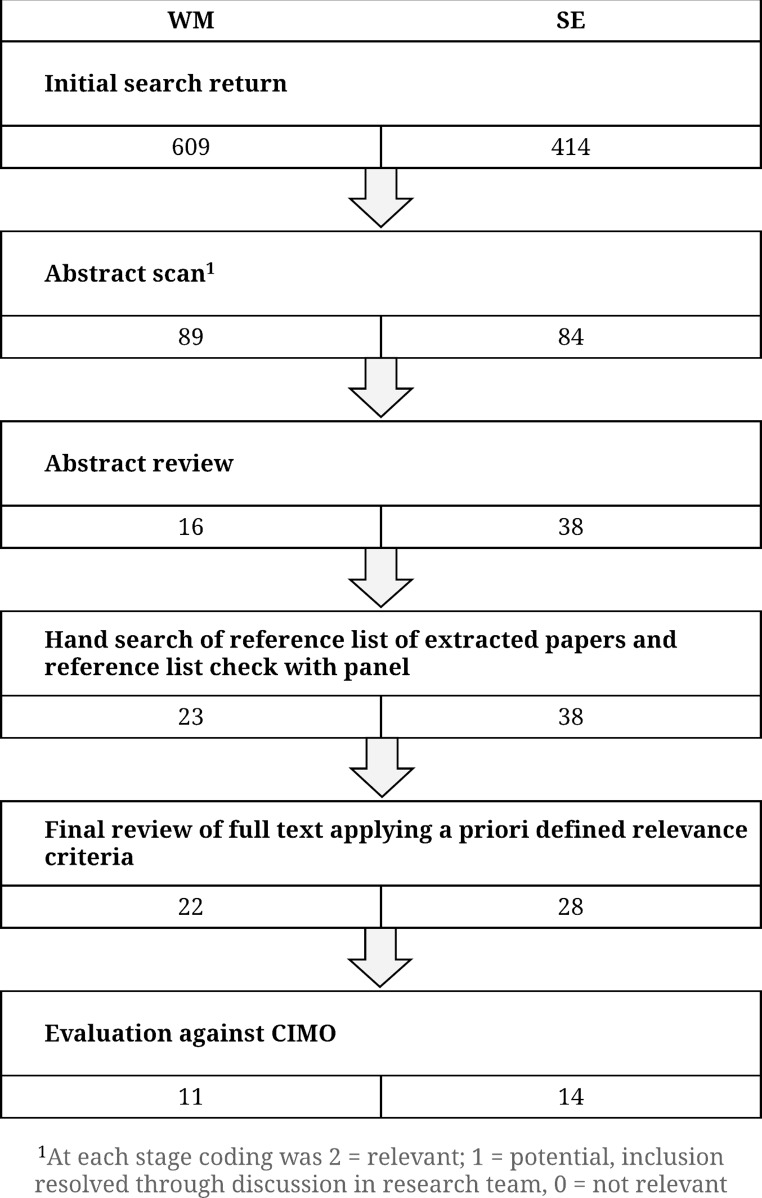
The iterative sifting process and the number included at each stage.

Fig 1 outlines the screening process at each stage. Some book abstracts were included initially if the topic was of high relevance and reviewed for primary sources which had not appeared in the EBSCO search. These activities returned a further fifteen papers for WM, of which four were included as relevant and none for SE. 22 WM studies and 28 SE studies were retained following abstract sifting and went forward for a systematic relevance and quality review of the full paper, cross-referenced between the authors, described in Fig 1.

### Relevance check

As recommended by Denyer and Tranfield’s [59] CIMO framework, we prioritized relevance criteria for Intervention and Mechanism; yet persistent lack of detail regarding the interventions rendered this difficult as, for instance, ‘training’ was used interchangeably to refer to computer-guided adaptive practice and face-to-face learning in education research. In ADHD research, many interventions considered themselves ‘psycho-social’ or ‘coaching’ but were actually interventions that targeted teachers’ or parents’ behavior and not coaching of the individual. Four authors were contacted via cited email and Research Gate to provide more information, but no responses received. The relevance scoring criteria are shown in Table 3. All papers with intermediate relevance were discussed between the researchers, including those using child samples, which we included based on consensus with the expert panel and in light of WM literature, which suggests differential effects for congenital WM deficit compared with acquired deficits [116]. Specific Learning Difficulties (SpLD) samples were thus required to assess point of principle and only child-based studies were available [77], [117].

**Table 3:** Relevance criteria.

CIMO and score	Value	Description		
**Context**	**3**	Adult, dyslexic, working population		
	**2**	Dyslexic or working		
	**1**	Adult only or child-based and dyslexic		
	**0**	Child, non-specified dyslexia		
**Intervention**	**3**	Coaching intervention specified, method of coaching clearly described and pedagogically dialectic		
	**2**	Face-to-face learning, methods clearly described		
	**1**	Face-to-face learning, not well described in terms of methods		
	**0**	Intervention not based on face-to-face learning–e.g. rTMS, asynchronous e-learning or self-study		
**Mechanism**	**3**	Working memory targeted, a reliable testing method clearly described	**3**	Self-efficacy targeted, a reliable testing method clearly described
	**2**	Working memory tested method for analysis inadequately described or blended with other measures	**2**	Self-efficacy tested method for analysis inadequately described or blended with other measures
	**1**	Other forms of memory targeted	**1**	Other forms of efficacy, esteem, confidence or agency targeted
	**0**	Working memory not addressed in this study	**0**	Self-efficacy not addressed in this study
**Outcome**	**3**	Work-related performance addressed and measures robust		
	**2**	Work-related, measures self-report alone		
	**1**	Adult-related success measures, not necessarily work (e.g. HE study; desistance etc.)		
	**0**	Unrelated to work or adult measures of success (e.g. word recognition or mental arithmetic)		
**Overall Score**	**I/M =**	4–9, plus C/O 4–6 Highly relevant, must include		
	**I/M =**	4–9 Good relevance for Realist synthesis, include		
	**C/O =**	4–6 Good relevance for narrative review of the field, include but separate		
	**I/M =**	2–3 Consider inclusion based on C/O score and quality of paper (must be raised with co-reviewer)		
	**I/M =**	0–1 do not include		

For SE, we excluded several studies after in-depth reading as they were either (1) highly specialized intervention targeting a context too far removed from target populations (for example, SE in nutrition of breast-feeding mothers in under-developed countries (2)) an intervention, though face-to-face, solely based on knowledge transfer and education not interactive and dialectic; and (3) SE not a target variable.

To reduce the risk of sampling bias, all extracted references were sent to the expert panel for independent checking and three responses were received (including one renowned WM expert from a UK research centre), all of whom confirmed the relevance of our extraction (including the child samples). One paper was added at this time.

### Quality check

Quality criteria were based on Rojon et *al*. [118] with an additional criterion of active bias management. The intermediate studies scored poorly due to their data analysis techniques; issues included: (1) reporting non-significant results using data sets without sufficient statistical power for the analysis used (e.g. using MANOVA with samples of 25 and four variables) and; (2) using multiple tests (ANOVA) without appropriate omnibus tests or corrections applied. This was noted for consideration in synthesis to qualify inferences made from the primary data and led to our decision to check and calculate effect sizes to assess impact, rather than rely on authorial conclusions.

One WM study [119] was excluded for failing to include an appropriate WM measure post-intervention. The final extraction included seven studies of high quality [77], [120–125] and three of intermediate quality [126–128]. A high-quality paper [77] was analyzed as two studies because it included two data sets that measured the same outcomes but using separate samples and interventions.

One SE study [129] was excluded for failing to establish at what point the post-intervention data were collected and not presenting sufficient detail on baseline measures. A second study [130] was excluded for not including SE as a dependent variable. Fourteen studies were retained, including eight studies of high quality [114], [131–137] and six of intermediate quality [138–143].

## Realist synthesis and study findings

We employed a common analytic strategy but synthesized the primary studies separately for WM and SE, comparing by (a) reviewing the effect sizes reported in each study; (b) grouping the studies according to outcome and; (c) examining contexts and interactions to identify any common themes. Where effect sizes had not been reported in the original paper (denoted by asterisk), these were computed from the means and standard deviations or *t*-test statistic and degrees of freedom as appropriate. A direct comparison between ‘successful’ (i.e., an observed effect in the expected direction) and ‘unsuccessful’ interventions allowed us to isolate effective principles across studies that could contribute to the design of a dyslexia coaching intervention protocol.

In the WM studies, the mean population ages ranged from 7.3 to 75 years; the studies used a variety of WM measures, all standardized and previously validated (references in Table 4 [117], [144–152]). Where effect sizes are noted with * they have been calculated by the author, not present in the original paper.

**Table 4:** Data extracted from working memory studies.

Author	Context			Interventions		Mechanism	Outcomes			
	Nationality, sample size, % female	Setting: Health / work / education / experimental group	Neurodeficit or identified WM deficit	Teaching / learning methods used	Time spent in intervention	Learning / psychological theories applied	Effect size of WM measure		WM test used	WM test Published by
**Alloway and Warner (2008)**	UK, 20, 45%	Education	100% Developmental Coordination Disorder, a condition that overlaps in presentation and symptoms with dyslexia [40]	Physical, group-based coaching to perform fine and gross motor tasks	65 x 1 hour	WM impact on learning	*d =* 0.97	Large	Verbal & visuo-spatial	[117]
**Ariës et *al*. (2014) study 1**	Holland, 92, 62%	Education	not known (n/k)	Computerized n-back practice and IMPROVE with group peer coaching to learn Metacognition (MC)	50 mins x 5 weeks	WM impact on learning, metacognition	*r* = .65	Large	n back & odd one out	[144] [145]
**Ariës et *al*. (2014) study 2**	Holland, 63, 54%	Education	n/k	Peer coaching to learn MC (no WM practice)	50 mins x 5 weeks	WM impact on learning, metacognition	*d = 0.89	Large	n back & odd one out	[144] [145]
**Chambers, Lo and Allen (2008)**	Australia, 20, 45%	Experimental	n/k	Mindfulness workshops	10-day course	EF, attentional control spotlight theory	*d = 0.52	medium	Digit span backwards only	[146]
**Craik et *al*. (2007)**	Canada, 49, 55%	Health	Age-related WM deficit	Group training knowledge transfer with practice and de-briefing	4 sessions	WM impact on learning	*d = 0.1	<small	Alpha span test	[147]
**Jha, Stanley, Kiyonaga, Wong and Gelfand (2010)**	US, 60, n/k	Work	Experimental group likely to be high ND % due to military role +stress	Mindfulness workshops plus coaching	24 hr total over 8 weeks	Cognitive control	Cannot calculate		Ospan	[148]
**Miranda, Presentacion, Siegenthaler and Jara (2013)**	Spain, 42, 14.8%	Education	ADHD	Small group dialectic workshops, in addition to parent/teacher interventions	16 sessions of 45 mins	WM impact on learning, self-regulation	*η^2^* = .125	Medium	WM sentences Digit span	[149] [150]
**Moro et *al*. (2012)**	Italy, 30, n/k	Health	Mild cognitive impairment (MCI) age-related	Cognitive training with personalized follow-up to coach strategies	6 months—2 month intensive 4 months weekly + practice	Metacognition	*d = 0.8	Large	Listening span test	[151]
**Moro et *al*. (2015)**	Italian, 30, n/k	Health	(MCI) age-related	Cognitive training with personalized follow-up to coach strategies	6 months—1 month intensive 5 months weekly + practice	Metacognition	*d = 1.28	Large	Listening span test	[151]
**Zeidan, Johnson, Diamond, David and Goolkasian (2010)**	US, 63, 60%	Education	n/k	Facilitation meditation workshop	4 sessions	stress management	Cannot calculate	N/A	Digit span backwards only	[152]
**Zylowska et *al*. (2008)**	US, 32, 62.5%	Experimental	ADHD	Small group mindfulness workshop	8 sessions of 2.5 hours	WM impact on learning, self-regulation	*d = 0.1	<small	Digit span	[152]

In the SE studies, the mean population ages ranged from 18 to 50 years. Many SE studies used a published General SE Scale (GSES, *n*-6); others used Teacher SE (*n*-2), specifically-constructed scales (*n*-2), Academic SE (*n*-1) and Study Skills SE (*n*-1). One study did not specify the scales used (*n*-1; [143]) but clarified that it was created with reference to the SCLT. One study included a GSES measure as well as a Job SE measure [133].

### WM synthesis

Table 4 shows the primary WM extraction.

#### WM improvement

Table 5 depicts effect sizes for WM improvement presented in numerical categories to ease comparison: (1) to indicate a small; (2) for medium and (3) for large effects. These calculations do not encompass sample size weighting but ease the process of qualitative interpretation required for narrative review. The extraction average of 2.1 suggested aggregate weighting in the medium range. The effect sizes of the successful studies ranged from medium to large, with only one statistically-significant study [121] falling short of medium effect size correlation (*r* = .25). Four studies reported non-significant results and the two effect sizes that were calculable for these were below the small range, as shown in Table 5. The effect sizes for successful studies were similar to the moderate aggregate effect sizes achieved for WM improvement through adaptive, computerized training, as reported in meta-analytic studies (*g =* .31, [153]; *g =* .35, [86]), indicating a trend towards similar levels of improvement for cognitive measures of WM resulting from a face-to-face intervention compared to computerized delivery protocols. Intervention context was not found to ‘matter’ for improvement of cognitive working memory at the aggregate level of analysis.

**Table 5:** Comparison of WM-specific and contextually-related dependent variables.

Author	Research design	Significance level of WM measure	Effect size of WM measure		Most workplace relevant contextual outcome selected and reported here	Effect size of contextual measure	
**Alloway & Warner, 2008**	Within contrasts repeated measures ANOVA	*F*(1,18) = 6.08, *p* = .02	*d =* 0.97	3	Reading and numerical test scores	Cannot calculate	
**Ariës et *al*. 2014 study 1**	Within/Between ANOVA at each interval, group comparisons from final test presented here	*F* (1,89) = 31.759, *p* = < .001	*r* = .65	3	Reasoning abilities test scores (second interval)	*r* = .13	1
**Ariës et *al*., 2014 study 2**	Within/between ANCOVA, metacognitive training versus control at final test presented here	*NS* due to Bonferroni corrected *p* value	*d = 0.89	3	Reasoning abilities test scores within-groups comparison	*r* = .38	2
**Chambers et *al*. 2008**	Within /between repeated measures ANOVA; Interval 2 control and intervention comparisons presented here	*F*(1, 39) = 7.81, *p* = .01	*d = 0.52	2	Mindfulness Awareness	*d = 0.25	1
**Craik et *al*., 2007**	Between-groups ANCOVA	*NS* Means and SDs reported	*d = 0.1	0	Secondary Memory (Logical Stories)	*d = 0.66	2
**Jha et *al*., 2010**	Within-groups (military trained) comparison paired samples *t-*test	Significant only with those reporting high practice, correlation between practice level and WM increase was *r* = .37, *p* = < .05	Cannot calculate for high practice groups only as M and SDs reported for all training groups		Positive and Negative Affect respectively–NB only Intervention group Means and SDs provided	*d = 0.5	2
**Miranda et *al*., 2011**	Between-groups ANCOVA (baseline scores as control variable) post intervention scores comparison reported here	*F*(1, 41) = 5.558, *p* = .024	*η^2^* = .125	2	Attention Vigilance test	*η^2^* = .288	3
**Moro et *al*., 2012**	Within pre-post (T1-T3) *t*-test reported as significant for intervention group A, effect size calculated from between-groups comparison at T2 for consistency, where group B act as a control group	*t*(14) = 2.48, *p* = .027	*d = 0.8	3	Attention–verbal span test selected as best work- related measure, again T2 between-groups comparison selected	*d = .84	3
**Moro et *al*., 2015**	Within pre-post (T1-T2) *t*-test reported as significant for intervention group A, effect size calculated from between-groups comparison at T2 for consistency, where group B act as a control group	*t*(14) = 2.3, *p* = .037	*d = 1.28	3	Montreal Overall Cognitive Assessment was selected as best work-related measure, again T2 between-groups comparison selected	*d = 1.08	3
**Zeidan, et *al*., 2010**	Within/between ANOVA, session x group reported here	*F*(1, 47) = 1.26, *p* = .27	Cannot calculate		Fatigue	*d = 0.7	3
**Zylowska et *al*. 2008**	Within-groups comparison only	*t*(24) = 0.45, *p* = .66	*d = 0.1	0	ADHD symptoms	*d = 0. 7	2
**Average effect size**				**2.1**			**2.7**

#### Intervention analysis

The interventions were typically a series of sessions conducted by either a professional in education/health or a trained meditation facilitator; some additionally utilized peer learning, although we note that facilitator type did not affect results. The number of sessions varied, with as many as 65 delivered over one year [120], yet as few as four for two of the studies [122], [125], the latter both notably reported no impact of their intervention on WM.

The methods review revealed consistent activities in the successful studies which could be interpreted to represent the four critical elements of Social Cognitive Learning Theory (SCLT: [66]): (a) verbal persuasion (an introductory knowledge transfer in most cases); (b) role-modelling (either current/past case study discussion or active role play); (c) vicarious learning (group discussion) and (d) mastery (the opportunity to practice or rehearse in context, with recognition of success). In unsuccessful studies by Aries ([77]: study 2, metacognition group only), Craik et *al*., and Zeidan et *al*. [122], [125], a lack of fidelity to SCLT resulted from an insufficient amount of time available to develop mastery and/or engage socially and these studies showed no improvement, again indicating the salience of a SCLT-compliant protocol. However, Zylowska et *al*. [124] provided sufficient time and discussion for all four SCLT steps, yet no improvement was noted in their study either. Following further in-depth review of the deviating paper, we noted that improvement in cognitive skills was not a stated outcome, nor did the protocol stipulate specific practice of metacognition or stress management, indicating the potential additional salience of such mechanisms which we now explore further.

‘Metacognition’, or the development of self-awareness in thinking [154], was mentioned explicitly in four successful studies ([77] study 1 & 2; [126–127]) and by association with similar terms in two others (‘attentional control’ [121] ‘cognitive control’, [128]. Becoming aware of and deliberately manipulating thoughts to improve memory has some support in the literature on dyslexia [4], [76]. Additional support is found in clinical dementia, educational and memory-specific research, in which ‘meta-memory’ (the ability to consciously be aware of and control mental memory tasks, such as visualizing a shopping list [155]) is improved through developing mental strategies and focusing on memory-related SE [116], [156–160]. The extracted studies support metacognition as a potentially viable psychological pathway for increasing WM capacity.

Two studies developed general self-awareness and metacognitive experience (as opposed to meta-memory specific) through mindfulness and meditation protocols [121], [128], reporting an increase in WM concurrent with a decrease in negative emotions (negative affect and stress, respectively). Since *increases* in anxiety and stress are known to reduce WM capacity [161], [162], the synthesis indicates an argument for *reductions* of stress as a moderating variable. Such insight is again further supported by dementia research [163] and relevant to our target population since research indicates stress management as problematic for dyslexic employees [74]. General mindfulness, unlike the targeted development of meta-memory, might not directly mediate improved WM but could potentially act via the moderating effect of reduced stress.

In short, when comparing intervention protocols between successful and non-successful studies in detail, we found that context did matter. The synthesis elicits that interventions supporting the development of memory-specific SE (through SCLT-compliant activities) may also result in the improvement of cognitive WM, which aligns with the clinical literature [95]. The social, metacognitive and emotional experience of participants points to the potential importance of high-fidelity training environments, as predicted by extant literature on training transfer in general [113] and the original developmental work of WM researchers [164], [165]. This finding was obscured by whole group aggregate effect size comparison and is in contrast to interventions involving practice divorced from environmental, social and emotional context (e.g. computerized WM training games). We now turn to the functional measures.

#### Functional outcome measures

**Fig 2:**
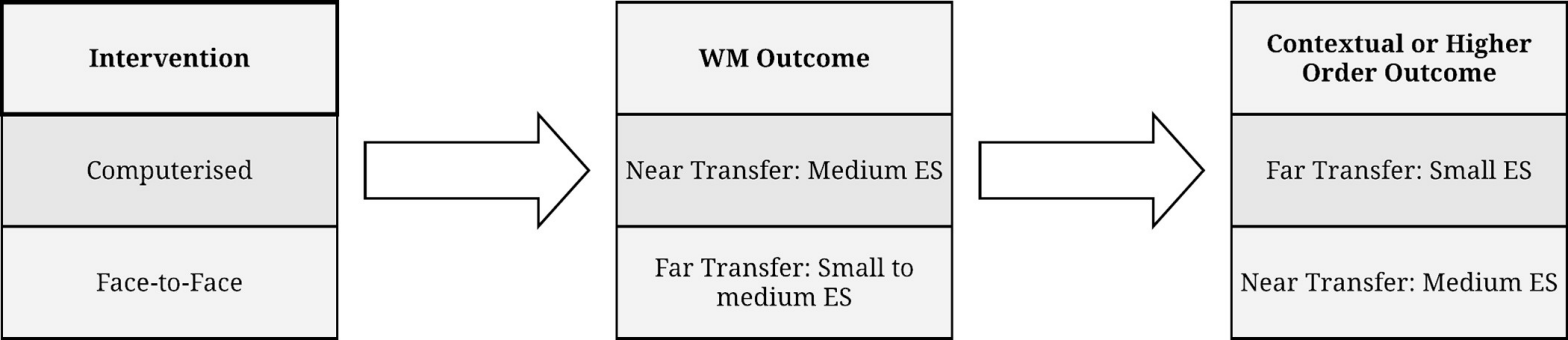
Comparison of near and far transfer effect sizes for computerized and face-to-face interventions.

The functional outcomes were heterogeneous in nature but consistent in improvement with medium effect sizes on average and appropriately significant *p* values were reported for all studies. This is in contrast to changes in WM scores which included four unsuccessful interventions. The computed aggregate effect size was 2.7, towards the top of the medium range, indicative of a stronger intervention effect than for WM measures. This is divergent from the computerized brain-training paradigm, where contextualized functional measures are typically weaker than the WM effects (*g* = .24, [86]; *g* = .20, [153]). Our synthesis suggests a reverse pattern for the present extraction as shown in Fig 2.

As observed across several studies [120], [121], [126–128], some contextualized training appears to improve core WM without direct practice of WM tasks; equally some WM computerized training improves functional measures without contextual training [144]. Of particular note is the second study by Aries et *al*. [77], where a contextual-based intervention resulted in functional comprehension improvements that did not overlap with targeted WM improvements. Coupled with extant WM research, this finding seems to indicate that while WM and functional outcomes may be related, the causal direction and transfer pathways between them are neither clear nor reliable.

### SE synthesis

Table 6 shows the SE extraction. Alpha co-efficients for scale reliability are presented in Table 6 (referenced in table where the scale was validated in a previous study,[166–168]).

**Table 6:** Data extracted from self-efficacy studies.

Author	Context			Intervention			Mechanism	Outcome	
	Nationality, sample size, % female	Setting: Health / work/ education /experimental group	Neurodeficit or identified WM deficit	Teaching /learning methods used	Time spent in intervention	Reliability of SE measure, α and additional reference, where appropriate	Learning / psychological theories applied	Effect size of SE measure	Time spent in intervention
**Bell, Raczynski and Horne (2010)**	US, 50, NK	Working in education	Not known (n/k)	Group-based knowledge transfer and discussion	7 sessions	.94	SCLT—teacher efficacy	*d =* 0.5148	Medium
**Engin and Cam (2009)**	Turkey, 22, 100%	Working in health	n/k—but nursing is generally thought to include up to 10% dyslexia prevalence (Sanderson-Mann & McCandless, 2006)	Group-based knowledge transfer and discussion	5 sessions	.81	SCLT–SE, autonomy	*r =* 0.88	Large
**Franklin and Doran (2009)**	Australia, 52, 59%	Education	n/k	2 workshops followed by 4 paired peer coaching sessions	9 hours	.86; Cited in [166]	PAAL; SCLT—SE; incremental implicit person theory	PAAL group *d =* - 1.21; Self-reg group—*d =* .1.08	Large
**McDowall and Butterworth (2014)**	UK, 32, 75	Education transition to work	n/k	Group coaching—facilitated discussion	1 session	.78	Strengths-based coaching		
**McGonagle et *al*. (2014)**	USA, 59, 86%	Work	Chronic health conditions	1:1 phone coaching	6 sessions of 1 hour	GSES:.80-.89 cited in [167] Job SE .77-.83 cited in [168]	SCLT–SE, also transactional and conservation of resources models of stress	GSES: Partial *η2* .18; Job SE: Partial *η2* .09	Large Medium
**McDowall, Freemann and Marshall (2014)**	UK, 54, 65	Work	n/k	1:1 coaching, two intervention conditions	1 session	0.83	Appreciative enquiry; feedback intervention theory	*η2* = .24	Large
**Reed, Kennett, Lewis and Lund-Lucas (2011)**	Canada, 41, NK	Education	20%	Seminar group with mixed info transfer and group discussion	NK—> 6	.89, Cited in [166]	Learned resourcefulness	*d =* .81	Large
**Reif, de Vries, Petermann and Gorres (2013)**	Germany, 234, 80	Health	n/k	Seminar group with mixed info transfer and group discussion	8 x 90 mins	.76-.9	‘Psycho-education’	*η2* = .10	Medium
**Stensrud, Gulbrandsen, Mjaaland, Skretting and Finset (2014)**	Norway, 21, 29	GPs at work	n/k	Role-play and debrief	5 x 4 hours	0.94	SE	n/a	N/A
**Style and Boniwell (2010)**	UK, 93, NK	Experimental	n/k	Group discussion 1/3; peer coaching 1/3; self-reflection 1/3	6 sessions	.76-.9	Positive psychology	*d =* .55	Medium
**Tsai et *al*. (2011)**	Taiwan, 395, 98	Working in health	n/k—but nursing as a profession has a dyslexia prevalence up to 10% (Sanderson-Mann & McCandless, 2006)	Group training	1.5 hours	0.94	Not stated at all	*r* = .26	Small
**Tschannen‐Moran and McMaster (2009)**	US, 93, NK	Working in education	n/k	Small group coaching; 1:1 coaching; observational 'live' coaching	5.75 hours	0.9	SCLT	*r* = .24	Small
**Watt, Murphy, Pascoe, Scanlon, and Gan (2011)**	Australian, 118, 89	Studying nursing	n/k—but nursing up to 10% (Sanderson-Mann & McCandless, 2006)	“Structured learning program”	3 days	0.69	Weak but SCLT related	*d =* .*87*	Large
**Zwerver, Schellart, Anema and van der Beek (2013)**	Holland, 40, 50	Working as doctors	n/k	Info transfer, role play, feedback	n/k	.75-.86	Theory of planned behavior, attitude, social norms and SE model	*r* = .33	Medium

As shown in Table 6, eleven studies reported medium to large effects and three studies reported small effect sizes. This pattern compares favourably to studies on online training to improve SE, which generally show smaller effects (see systematic review: [169]. Eight studies included measures of academic- or work-related performance, which also significantly improved as a result of the intervention; this is as expected based on the reliable extant literature on SE and functional performance. We now analyze the primary studies in some depth to elicit principles relevant to workplace dyslexia coaching protocols.

#### Intervention analysis

The participants in the SE studies were typically adults engaged in learning related to their studies or work. The interventions tended to be delivered by a mix of professional educators and facilitators trained in a specific, work-related process (e.g., [114], [131]), although some studies additionally utilized peer-to-peer coaching without detailing their training [114], [131], [142]. Again, the type of facilitator did not affect the results.

Paralleling the WM synthesis, the successful interventions involved all four elements of SCLT, either overtly within the intervention structure or by allowing time for development of mastery before reassessment. In most cases, participants’ SE was developed in relation to a clear and measurable learning outcome or goal related to their work or life, rather than directly targeting SE; this approach is congruent with Bandura’s [66] original proposition. We thus observed successful interventions consistent with Goal Setting Theory (GST; [170]) in addition to SCLT. GST predicts that ‘goal clarity’ (GS1) focuses attention and inspires effort and persistence to achieve while creating the conditions for metacognition around the target behavior; this element was clearly adhered to in the extracted studies through the verbal persuasion element. However, GST further proposes two other moderators for improvements in work performance: (GS2) SE for achieving the goals and (GS3) the commitments made to others in relation to the goals and their social value, the extent to which they enhance the ‘Social Identity’ [171] of the individual learner. We noted sufficient attention to GS2 through mastery and rehearsal but considerable variability regarding GS3, which we inferred through the common occupational or educational contexts of each sample, with the exception of the study by Style and Boniwell [142]. We propose that SE is more likely to develop with positive, socially contextualized and internalized goals, as highlighted in generalist workplace coaching literature as important to organizational outcomes [111], [172] which are essential for successful disability accommodation. Again, the context mattered.

The time spent in interventions, typically shorter than those in the WM papers with the longest only five sessions of four hours, did not affect success, since even those with a single intervention session reported a significant impact on SE [131], [132]. The only study indicating no improvement [141] reassessed SE at the immediate end of the program before the participants could practice skills in their own setting (i.e., to develop mastery), thus potentially reflecting a methodological artefact. Indeed, Tsai et *al*. [143] observed that SE decreased in the period immediately after the intervention before recovering to an increase from baseline after three months, indicating practice time is needed to obtain mastery and/or that participants may undergo an internal process of re-evaluation and adjustment. In the single session interventions, mastery was addressed by asking participants to recall and explore incidences of previous mastery [131], [132], further corroborating that a practice/rehearsal/mastery element appears key for successful interventions targeting both WM and SE.

An active goal-setting component, which we inferred through the intervention description rather than through explicit statements in the studies with the exception of McDowall et *al*. [132], and the resulting level of cohesion between learning goals and socially interactive development of SE, facilitated consistently successful outcomes. This **is** consistent with coaching psychology literature [111], [173].

### Relevance to dyslexia coaching

We now revisit our previously stated concern about sample specificity, addressing whether, in principle, the findings of the synthesis could be applied to dyslexia coaching.

#### Population

The search protocol included a broad age range (from 7 to 75 years). All SE studies were based on working age adults and therefore directly comparable. Successful WM studies included wide age ranges demonstrating that, in principle, WM improvement is not contingent on age. Although the child sample-based WM studies [77], [120] were retained for condition similarity as advised by the panel, we note that, in one of these, the intervention protocol was substantially longer than other studies [120]; hence intervention length would be useful to consider as a moderator in subsequent dyslexic accommodation research. Participant gender was well-balanced across studies and did not affect any results.

#### Intervention protocol

Current practice in UK dyslexia coaching is to provide an average of 4–5 sessions delivered on a one-to-one basis [74]. Extracted studies that matched this structure were broadly successful; however, the quality of coaching and adherence to SCLT and GST may be as important as intervention time for facilitating improvement to both WM and SE. Further research should outline the *process* (not only the outcome) in more detail.

One divergence between the primary studies and dyslexia coaching practice is the use of group versus one-to-one training. Some studies included one-to-one elements [131–133], yet all intervention protocols except one [133] reported some element of group discussion, peer coaching or coaching triads. The one-to-one intervention achieved a positive result; however, a greater understanding of the group dynamics in WM and SE outcomes is needed. It is possible that a dyslexic population might benefit further from group coaching; the peer support element could improve the quality of the SCLT stages ‘vicarious learning’ and ‘role-modelling’, but no current studies have compared the impact of group coaching with one-to-one protocols.

## Discussion: Implications for research and practice

**Fig 3:**
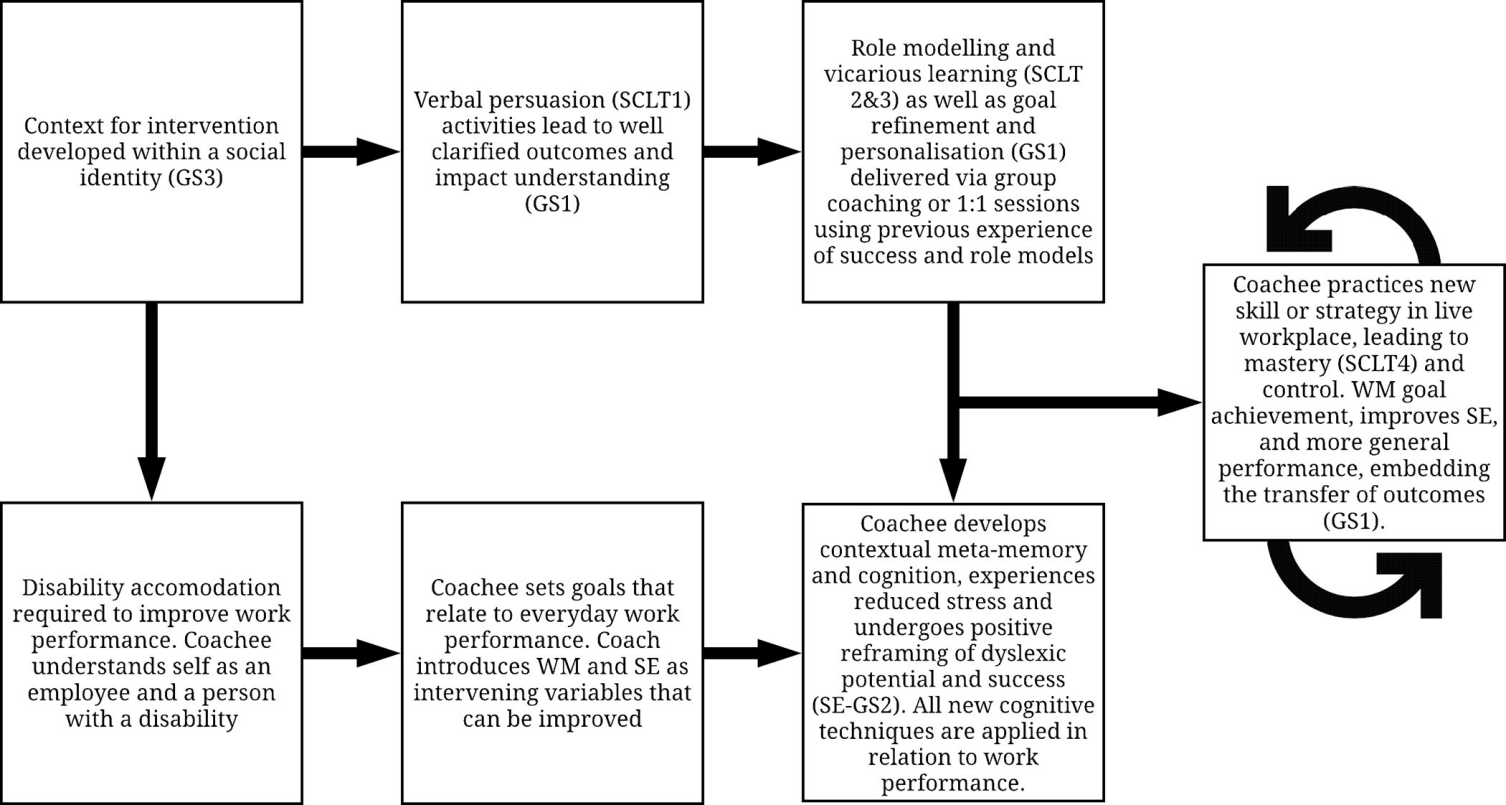
Hypothetical use of SCLT, metacognitive development and GST to improve SE & WM through face-to-face learning interventions.

We set out to investigate to what extent, and under what conditions, face-to-face learning interventions improve WM and SE, in turn leading to potential functional improvements in work performance. We found evidence of medium effect sizes for both mechanisms as well as for functional, work-related outcomes. We conclude that there is sufficient evidence that findings could be applied to dyslexia coaching protocols and highlighted the following key components, depicted in Fig 3. Fidelity to SCLT and GST protocols are important process conditions for the effectiveness of coaching to improve SE. Evidence that coaching improves WM was less consistent, but also contingent on fidelity to SCLT (in particular mastery experiences) combined with development of self-awareness and/or stress management through metacognitive practice. Such consistency in employing SCLT-compliant protocols across the WM and SE studies was surprising and indicated that any new skill, including development and management of cognitive function such as WM, is contingent on the individual learner’s social and emotional/metacognitive experience. Particular attention should be devoted to practice opportunities when developing a new skill and the opportunity to frame developments as ‘mastery’ in a social context. WM skills, which are of high concern to our target population, may not simply be ‘taught’; learners must develop them for themselves, with support and reflection on mastery opportunities. Such observations resonate with the clinical literature [95] and are aligned with a social identity perspective [171], which broadly holds that group membership and social context is important for individual behavior, identity and self-concept. Accordingly, we propose that future interventions should pay attention to the relevance of context in which behaviors are learned and practised.

The preliminary intervention protocol depicted in Fig 3 has been drawn from our synthesis and can be used as a guide for future research, as well as a theoretical basis for the design of group- and individual-based coaching activities. For example, GST is associated with many tried and tested practices such as the ‘GROW’ model in coaching [97]; the development of mastery is associated with the common training practice of reflection cycles [113], [174] and metacognitive development can be facilitated using techniques such as ‘Clean Language’ to elicit knowledge about self-help strategies [175]. We hope that practitioners will consider our theoretical pathway and reflect on its congruence with their own delivery tools.

Longitudinal analysis of impact, including job sustainability and promotion rates, will provide objective data that can be used to assess the effectiveness of interventions as a disability accommodation, given that such evidence remains sparse. Our review highlighted large geographical disparities linked to differences in legislation, organizational knowledge and the availability of trained professionals to support interventions for dyslexia; for example, we did not find evidence of coaching deployed in workplaces outside the UK. As we deliberately excluded grey literature to isolate research-based evidence regarding objectively-measured psychological variables to guide contemporary practitioner guidance [71], [72], further assessment of practitioner evidence may further the field.

### Functional improvements: Context matters

Coaching is a viable activity for SE improvements as both process mechanism and outcome, which is congruent with the wide-ranging literature on the development of SE as a facilitator of workplace and educational performance [111]. Our synthesis added value in drawing out the contextual intervention features that supported successful delivery, including GST.

Coaching for WM improvements was less clearly effective for both mechanism and outcome variables compared to the SE studies, as demonstrated by the overall lower effect sizes. In particular, the second study by Aries [77] indicated that WM was somewhat decoupled from contextual performance such as comprehension, contrary to accepted theory that WM underpins complex thought [65]. While strong WM capacity as a contingent factor for performance across education and work has been well documented (see introductory sections), any assumption that improvements in WM will therefore *mediate* improvements in higher-order, functional skills was not demonstrated by our synthesis or in the extant literature; an observation echoed by some contemporary WM authors [85], [176], [177].

Our analysis supports the proposal that, even when low WM may have created a functional performance problem, WM improvement may not be the only or most effective route to performance improvement [116]. Our synthesis of ‘contextually congruent’ interventions (i.e. face-to-face and sharing functional goals rather than computerized) elicited stronger evidence for functional performance than cognitive performance; a reverse effect to studies using primarily computerized training (as in Fig 2). We contend that coaching can facilitate contextualization specific to the person and their immediate environment, where coach and coachee co-create functional goals. These may then be addressed through behavioral, emotional and social mechanisms rather than solely focused on increasing core WM capacity, hence the decoupling of the espoused relationship between WM and performance. This observation is consistent with research on training transfer [113], [178] and indeed the early work of WM researchers on memory encoding specificity [164], [165]. We posit that congruence in the contextualization of interventions with mechanisms and outcomes is a crucial factor for future dyslexia coaching studies and indeed in WM research more widely.

## Conclusion

Drawing on established theories, we found that increased socio-cognitive competence and confidence, concurrently with enhancement of metacognitive skills improved functional outcomes. Our propositions are put forward as a ‘pump-priming’ exercise, given the small number of primary studies and the nascent research agenda [179]. Context matters for developing functional skills and thus coaching is a viable intervention, bringing greater ecological validity for the work performance improvement of dyslexic adults than literacy-based or computerized interventions. The strength of this narrative review is the identification of a conceptual intervention framework to set the agenda for potential psychological mechanisms that may facilitate increased work performance for dyslexia, through coaching as a reasonable disability accommodation for dyslexic employees. As employers require evidence-based guidance to accommodate work for a potentially vulnerable, significant minority of employees, psychological research must now test and build upon our conceptual framework to address the evidence blind spot.

## Supporting information

S1 File PRISMA checklist coaching dyslexia at work. (PDF)
